# Age Moderates Associations Between Helping and a Sense of Meaningfulness in Daily Life

**DOI:** 10.1093/geroni/igaf122.204

**Published:** 2025-12-31

**Authors:** Enna Chen, Claire Growney, Laura Carstensen

**Affiliations:** Stanford University, Stanford, California, United States; Stanford University, Stanford, California, United States; Stanford University, Stanford, California, United States

## Abstract

The aim of the present study was to examine whether helping others in daily life is associated with meaning in life and experienced emotions, and whether age moderates the associations. A sample of 180 individuals aged 18 to 93 years participated in an experience sampling study, where they reported their emotions, experience of meaning, and whether they were engaging in helping behavior in the moment at five randomly selected times per day for seven days. Averaging across the week-long period, age was positively associated with helping frequency. Individuals who helped others more often throughout the week reported more meaning in life. Age did not moderate this association. However, within-individual analyses suggested that when participants were helping others, they experienced higher levels of meaningfulness compared to other activities; and this relationship was stronger in younger than older participants. At the between-person level, helping was also associated with more positive emotions (and unrelated to negative emotions); this effect was stronger for older than younger participants. Despite this, at a within-person level, helping others predicted a higher level of momentary negative emotions (but not momentary positive emotions) regardless of age. Findings suggest that older adults help others more than younger adults in daily life, which is consistent with a small but growing literature suggesting that prosociality increases with age. While individuals experienced more negative emotions while helping, the tendency to help is related to reporting higher mean levels of positive emotions and a greater sense of meaningfulness.

